# Table tennis players use superior saccadic eye movements to track moving visual targets

**DOI:** 10.3389/fspor.2024.1289800

**Published:** 2024-02-09

**Authors:** Riku Nakazato, Chisa Aoyama, Takaaki Komiyama, Ryoto Himo, Satoshi Shimegi

**Affiliations:** ^1^ Graduate School of Frontier Biosciences, Osaka University, Toyonaka, Osaka, Japan; ^2^ Graduate School of Medicine, Osaka University, Toyonaka, Osaka, Japan; ^3^ Center for Education in Liberal Arts and Sciences, Osaka University, Toyonaka, Osaka, Japan; ^4^ Faculty of Science, Osaka University, Toyonaka, Osaka, Japan

**Keywords:** catch-up saccade, moving target, virtual reality, ball sports, table tennis

## Abstract

**Introduction:**

Table tennis players perform visually guided visuomotor responses countlessly. The exposure of the visual system to frequent and long-term motion stimulation has been known to improve perceptual motion detection and discrimination abilities as a learning effect specific to that stimulus, so may also improve visuo-oculomotor performance. We hypothesized and verified that table tennis players have good spatial accuracy of saccades to moving targets.

**Methods:**

University table tennis players (TT group) and control participants with no striking-sports experience (Control group) wore a virtual reality headset and performed two ball-tracking tasks to track moving and stationary targets in virtual reality. The ball moved from a predetermined position on the opponent's court toward the participant's court. A total of 54 conditions were examined for the moving targets in combinations of three ball trajectories (familiar parabolic, unfamiliar descent, and unfamiliar horizontal), three courses (left, right, and center), and six speeds.

**Results and discussion:**

All participants primarily used catch-up saccades to track the moving ball. The TT group had lower mean and inter-trial variability in saccade endpoint error compared to the Control group, showing higher spatial accuracy and precision, respectively. It suggests their improvement of the ability to analyze the direction and speed of the ball's movement and predict its trajectory and future destination. The superiority of the spatial accuracy in the TT group was seen in both the right and the left courses for all trajectories but that of precision was for familiar parabolic only. The trajectory dependence of improved saccade precision in the TT group implies the possibility that the motion vision system is trained by the visual stimuli frequently encountered in table tennis. There was no difference between the two groups in the onset time or spatial accuracy of saccades for stationary targets appearing at various positions on the ping-pong table.

**Conclusion:**

Table tennis players can obtain high performance (spatial accuracy and precision) of saccades to track moving targets as a result of motion vision ability improved through a vast amount of visual and visuo-ocular experience in their play.

## 1 Introduction

In ball sports, such as table tennis and baseball, physical actions, such as ball hitting, are executed and adjusted according to the visual information of the ball moving in three-dimensional space. The motor control based on this information is referred to as visuomotor control. Visuomotor performance is determined by the quality and quantity of the visual motion information, which greatly depends not only on the visual system that processes the visual information but also on the gaze control system that acquires the information. Accordingly, research has investigated whether eye movements used by athletes are superior to those of non-athletes.

The way ballgame players move their eyes to collect information during play is referred to as “gaze behavior”, to which head and eye movements contribute (1–4). Various gaze behaviors are performed depending on each ball sport, but basically, they consist of a combination of two representative eye movements: saccade and smooth pursuit (5). Smooth pursuit eye movement enables tracking a moving target smoothly and continuously but has the limitation of the eye movement velocity, where continuous ocular tracking becomes difficult if the target speed exceeds 60 to 70 deg/s. To compensate, the gaze is directed by saccades from the current gaze position to the future target position (3).

In table tennis, eye movement plays a greater role than head movement in gaze behavior when directing the eye toward the ball (4). Furthermore, saccades track the ball since the speed of a ping-pong ball often exceeds the threshold of smooth pursuit (6, 7). When table tennis players track a ball coming toward them, players gaze at the opponent's racket striking the ball and direct their gaze to the ball after the strike (catch-up saccades) (6). Based on the information obtained there, players predict where the ball will land and shift their gaze there before the ball arrives (predictive saccade) (8). Therefore, the spatial accuracy of the saccade determines the quality of the ball motion information, which in turn affects the aiming performance of the racket (6, 8). Thus, better saccade ability is expected to be associated with better play.

Several studies have compared saccade ability between various ball athletes including table tennis and non-athletes, showing that athletes have shorter saccade latency (time from stimulus presentation to saccade onset) than non-athletes (9–11). On the other hand, other studies (12, 13) did not find a difference in saccade latency between such groups. These studies included different types of sports athletes and different measurement conditions, so the cause of the discrepancy in results is unclear. Since almost all studies used stationary stimuli, it is possible that the ball players' potential saccadic abilities were not brought out. Additionally, spatial accuracy (14), which is extremely important for ball players' saccadic performance, has rarely been measured. To clarify this point, saccadic ability not only for stationary targets but also for moving targets should be evaluated at least, and in addition to the saccade onset time, the speed and spatial error at the endpoint, etc., should be comprehensively investigated.

When the visual system is exposed to a specific visual stimulus for a long time or frequently, its detection sensitivity to the exposed stimulus improves through plastic changes called perceptual learning (15). For example, prolonged motion stimulation has been reported to improve perceptual motion detection and discrimination abilities specifically in the stimulated motion direction (16–19). Therefore, ball athletes may have an improved ability to analyze the motion of moving targets they often see during play and to predict their trajectories and future destinations. If so, this may also contribute to the improved spatial accuracy of body movements. Since ball athletes track the ball to obtain accurate information during play, investigating their ball-tracking ability is effective as a powerful probe to derive the visual improvement effect and the influence on physical movement. We focused on table tennis, which provides an overwhelming amount of visual motion stimuli compared to other sports, and the balls are tracked mainly by saccades. The potential advantage of saccadic ability in table tennis players may be revealed by using a ball that moves in the depth direction as seen in normal table tennis scenes.

Therefore, we hypothesized that table tennis players will be better able to analyze the motions of moving targets they frequently see during play and predict their trajectories and future destinations accurately, improving the spatial accuracy of saccades that capture moving targets. This study aimed to verify this hypothesis by comparing the saccade ability between table tennis players and non-athlete controls from the perspective of differences in saccade targets, i.e., moving vs. stationary balls and familiar vs. unfamiliar trajectories for moving balls. We assumed that table tennis players would show a superior saccade ability for moving targets over static targets, and for moving targets, for balls with familiar trajectories over unfamiliar trajectories.

In real table tennis, even if a table tennis expert throws out a ball, it is impossible to repeatedly release the ball under exactly the same conditions (trajectory, speed, and direction). We also needed to generate the motion of a real ping-pong ball and the motion of an unfamiliar ball trajectory at different speeds to assess whether there is a visual experience-dependent visual function improvement effect. In addition, ball tracking without a fixed head in a standing position is not suitable for the purposes of this study. This is because the movement of the head also contributes to the movement of the gaze in no small way and the movement of the head accompanying the disturbance of posture directly causes movement of the eyes. Therefore, we used virtual reality (VR) technology to accurately reproduce the trajectory of a ball in a situation close to an actual table tennis scene and measured the gaze movement due to eye movement by fixing the head.

## 2 Materials and methods

### 2.1 Experiment 1: moving ball-tracking task

#### 2.1.1 Participants

Healthy university students who had a history of playing table tennis for three years or more (six male and four female, age: 21.0 ± 1.18 years old) and university students who had no history of playing table tennis or other ball-hitting sports (five male and five female, age: 23.6 ± 3.74 years old) participated in the experiment as the table tennis player (TT) group and control group (Control), respectively. All participants underwent a visual acuity test using a Landolt ring, and all had confirmed binocular or corrected visual acuity of 1.0 deg or better. This study was approved by the Osaka University Human Ethics Committee (16,207) and complied with the standards set by the latest revision of the Declaration of Helsinki, except for registration in the database, and each participant provided written informed consent.

#### 2.1.2 Apparatus

**Figure 1:**
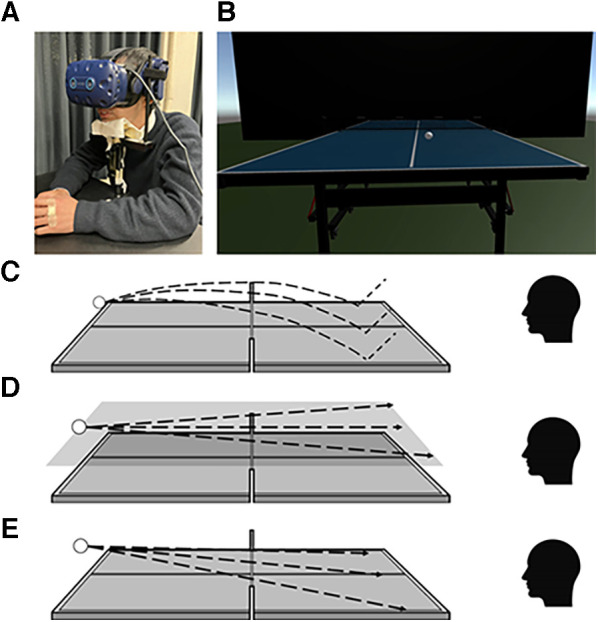
Schematic of the experimental setup and experimental conditions. (**A**) A participant performing a task with his head fixed. (**B**) Image actually seen by participants. (**C–E**) Ball trajectories under parabolic (**C**), horizontal (**D**), and descent (**E**) motion conditions.

The VR environment was designed on a computer containing an Intel Core i7–7,700 K, 4.2 GHz Processor, and Nvidia Geforce GTX 1080ti graphic card. Its output was connected to the VIVE Pro Eye headset (HTC Corporation, New Taipei City, Taiwan) via a link box (Figure 1A). The head-mounted display (HMD) can provide a resolution of 1,440 × 1,600 pixels (615 PPI) per eye with a refresh rate of 90 Hz and a field of view of 110 deg. The eye tracker built into the VR headset records the ocular movement at 90 Hz with an accuracy of 0.5–1.1 deg (20), and the measured data were stored in the computer storage. The center position of the spherical coordinate for VR was calibrated for each experiment as a room setup using pre-installed software. The gaze position in VR space was calibrated before each experiment using eye-tracking software.

#### 2.1.3 Visual stimulus

A visual stimulus was created in the VR space using the free software Unity. In the VR space (Figure 1B), a table tennis table (depth × width × height from the ground = 2.76 m × 1.575 m × 0.76 m) was set in front of each participant. The distance between the participants and the front end of the table tennis table was one meter. A black wall was set one meter further away from the far end of the table tennis table.

#### 2.1.4 Task and procedure

In an actual table tennis scene, a saccade is induced immediately after the opponent hits the ball (8). Therefore, the ball-tracking task asked participants to follow with a gaze as much as possible the ball moving from a starting point 30 cm above the far edge of the table tennis table toward the front side. In the task, three types of ball trajectories (parabolic, horizontal, and descent), three types of courses (left, middle, and right), and six speeds were combined to make 54 test conditions.

A parabolic ball motion condition was set as the “familiar ball trajectory” most frequently experienced by table tennis players. The parabolic motion was reproduced according to the data obtained by tracking and recording the movement of the ball, which was fired from a ball-injecting robot consisting of three rotating rotors onto the real table tennis table in the real world (Figure 1C). Next, we set a horizontal motion condition as an unfamiliar trajectory that moves in the horizontal direction with uniform linear motion; this motion does not occur in real table tennis. Under this condition, the ball keeps moving at the same height (30 cm) parallel to the table tennis table (Figure 1D). Finally, the condition for the ball to descend in a uniform linear motion was set as the descent motion condition (similar to a smash but as an unfamiliar trajectory). Here, the ball descends diagonally and linearly toward the points on the table surface which correspond to the bounce points in the parabolic motion condition (Figure 1E). The ball speed range was determined according to the average ball velocity during a rally in an actual table tennis scene (21), and we set the reference speed to be 6.0 m/s. At the reference speed, the arrival time of the ball from the start position to the end position was 0.33 s in the horizontal motion condition. The six-speed conditions (1.2, 2.4, 3.6, 4.8, 6.0, 7.2 m/s) were determined by multiplying the reference speed by the constants 0.2, 0.4, 0.6, 0.8, 1.0, and 1.2, respectively.

The participants sat on a chair with the HMD attached, and their heads were fixed on the chin stand. For this task, the ball was presented at the predetermined starting point on the HMD, started moving 3 s after the ball appearance, and then disappeared just after pausing at the endpoint of each course. The participants were instructed to keep their gaze on the ball presented as accurately as possible and to track it as the ball started to move. The period from the appearance of the ball to its disappearance was regarded as one trial. At the same time as the ball disappeared, a new ball was presented at the start position, and the next trial began with the same sequence. For each trial, the ball's trajectory, course, and velocity conditions were all randomly determined. One set consisted of 10 trials (10 balls), and one block consisted of six sets. The participants were able to rest for about 15 s between task sets. It took about 10 min to complete one block of tasks. To avoid fatigue, a 3-minute rest was provided between the blocks, and a total of six blocks were performed per day (360 balls in total) for 3 days. Participants were verbally asked whether they felt fatigued between blocks and asked if they needed to discontinue the experiment due to fatigue. As a result, no participants asked to cancel or postpone the experiment due to fatigue. Participants observed a total of 1,080 virtual balls, in which 20 trials per condition were conducted. A calibration of the gaze position measurement was performed between sets. The number of practice sessions for the ball-tracking task in Experiment 1 was six sets and was conducted on the first day of the 3-day experiment.

#### 2.1.5 Data analysis

The ball position, eyeball position, and gaze direction in all trials were recorded as coordinate positions and unit vectors. Using the data obtained from the right and left eyes, we analyzed the ball tracking accuracy. Comparisons between groups were made using the average of the right-eye data and left-eye data for each saccade parameter (occurrence rate, onset time, velocity, and endpoint error).

##### 2.1.5.1 Evaluation of ball position and gaze direction

**Figure 2:**
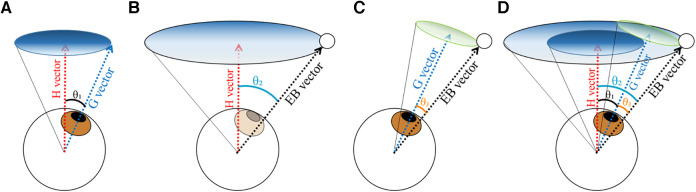
Schematic of the analysis of the ball position and gaze direction. (**A**) Gaze direction. The vector extending straight from the eyeball center perpendicular to the frontal plane was defined as the H vector, and the vector in the direction of the gaze as the G vector. The gaze direction was defined as the angle *θ*₁ between the G vector and H vector. (**B**) Ball position. The vector from the eyeball center to the ball center was defined as the EB vector, and the ball position was determined as the angle *θ*₂ between the EB vector and H vector. (**C**) Gaze errors. Angle *θ*3 between the G vector and EB vector was defined as the gaze error.

The ball position and gaze direction during the task were determined by calculating the relative angle from a reference vector. The reference vector (horizontal vector) was defined as a vector extending straight and horizontally forward from the center of the eyeball, assuming the situation that the participant was looking straight into the distance without controlling their extraocular muscles. The position data of the ball and gaze were measured separately into horizontal and vertical components and were integrated into a synthetic vector on spherical coordinates with the origin at the center of the eyeball. We defined the gaze direction (G vector in Figure 2A) as the angle *θ*₁ formed by the gaze direction vector and the reference vector. Similarly, the ball direction (EB vector in Figure 2B) was defined as the angle *θ*₂ formed between the vector extending from the eyeball to the ball and the reference vector.

##### 2.1.5.2 Evaluation of gaze direction with respect to the ball

To quantitatively evaluate how accurately the gaze was directed at the ball, angle *θ*_3_ formed by the eye-ball vector and the gaze direction vector was regarded as the spatial accuracy of the gaze direction (Figure 2C).

##### 2.1.5.3 Detection and evaluation of saccadic eye movement

The rotation speed of the eye movement was obtained by differentiating *θ*₁, and the time when the speed of *θ*₁ became 30 deg/s or more was defined as the saccade onset time. However, if the amplitude of *θ*₁ was 2 deg or less or the duration was 20 ms or less, it was excluded from the saccade (22). The time when the speed of *θ*₁ became 30 deg/s or less was defined as the saccade offset time. Angle *θ*₃ at the end of the saccade was called the saccade endpoint error and was used as an index of the spatial accuracy of the saccade. The smaller the saccade endpoint error, the more accurate the saccadic movement to the ball. Saccade precision (consistency and reproducibility of movements across trials) was calculated as the variation coefficient of the saccade endpoint error.

##### 2.1.5.4 Detection and evaluation of smooth pursuit eye movement

In addition to saccades, participants can follow the ball with smooth pursuit eye movements. Therefore, in trials in which no saccade was observed, eye movements that captured the target within a radius of 2.5 deg of the fovea for more than 90% of the total trial time were defined as smooth pursuit eye movements.

##### 2.1.5.5 Statistical analysis

The occurrence rate, onset time, speed, and saccade endpoint error were statistically analyzed by a two-way ANOVA with the sports group (TT and Control) and ball velocity (0.2, 0.4, 0.6, 0.8, 1, 1.2) as factors. If the interaction was significant, a *post hoc* test was performed for each speed condition using Bonferroni's multiple comparisons. Friedman's test was used to compare the variation coefficient of saccade endpoint error between the two groups. The level of significance for all the analyses was less than 5%. All data are expressed as the mean ± standard error.

### 2.2 Experiment 2: stationary ball-tracking task

#### 2.2.1 Participants

Healthy university students with more than 3 years of table tennis experience (nine male, one female, age: 20.7 ± 2.00 years old) and those who had no history of ball-hitting sports such as table tennis (eight male, two female, age: 21.4 ± 3.10 years old) participated in the experiment as the table tennis player group and control group, respectively. All participants had binocular or corrected visual acuity of 1.0 deg or better. This study was approved by the Osaka University Human Ethics Committee (16,207) and conformed to the standards set by the latest revision of the Declaration of Helsinki, except for registration in the database, and each participant provided written informed consent.

#### 2.2.2 Apparatus and visual stimulus

The experimental apparatus used in this experiment was the same as in Experiment 1. The visual stimulus was also presented in a VR space with a table tennis table and walls as in Experiment 1.

#### 2.2.3 Ball-tracking task

The participants were asked to direct their gaze at a ball appearing at a random position on the table tennis table.

#### 2.2.4 Task procedures

As in Experiment 1, the participants sat on a chair with a HMD and fixed their heads on the chinrest and were instructed to direct their gaze to the ball. In the task, a stationary ball was presented at a random position about 30 cm above the table tennis table. The ball disappeared after being presented for 3 s, and at the same time, the next ball was presented at a random position. One set consisted of 10 repetitions of ball presentation, and one practice set and three test sets were performed. The number of practice sessions for the ball-tracking task in Experiment 2 was one set and was conducted.

#### 2.2.5 Data analysis

As in Experiment 1, angle *θ*_3_ formed by the ER vector and the G vector after the saccade was regarded as the spatial accuracy of the saccade. Data from the right and left eyes were used for the analysis. Comparisons between groups were made using the average of the right-eye data and left-eye data for each index.

#### 2.2.6 Statistical analysis

The saccade error was compared between the table tennis player group and the control group using a one-way ANOVA. The significance level was set to less than 5% for all data. All data are shown as the mean ± standard error.

## 3 Results

### 3.1 Experiment 1

**Figure 3:**
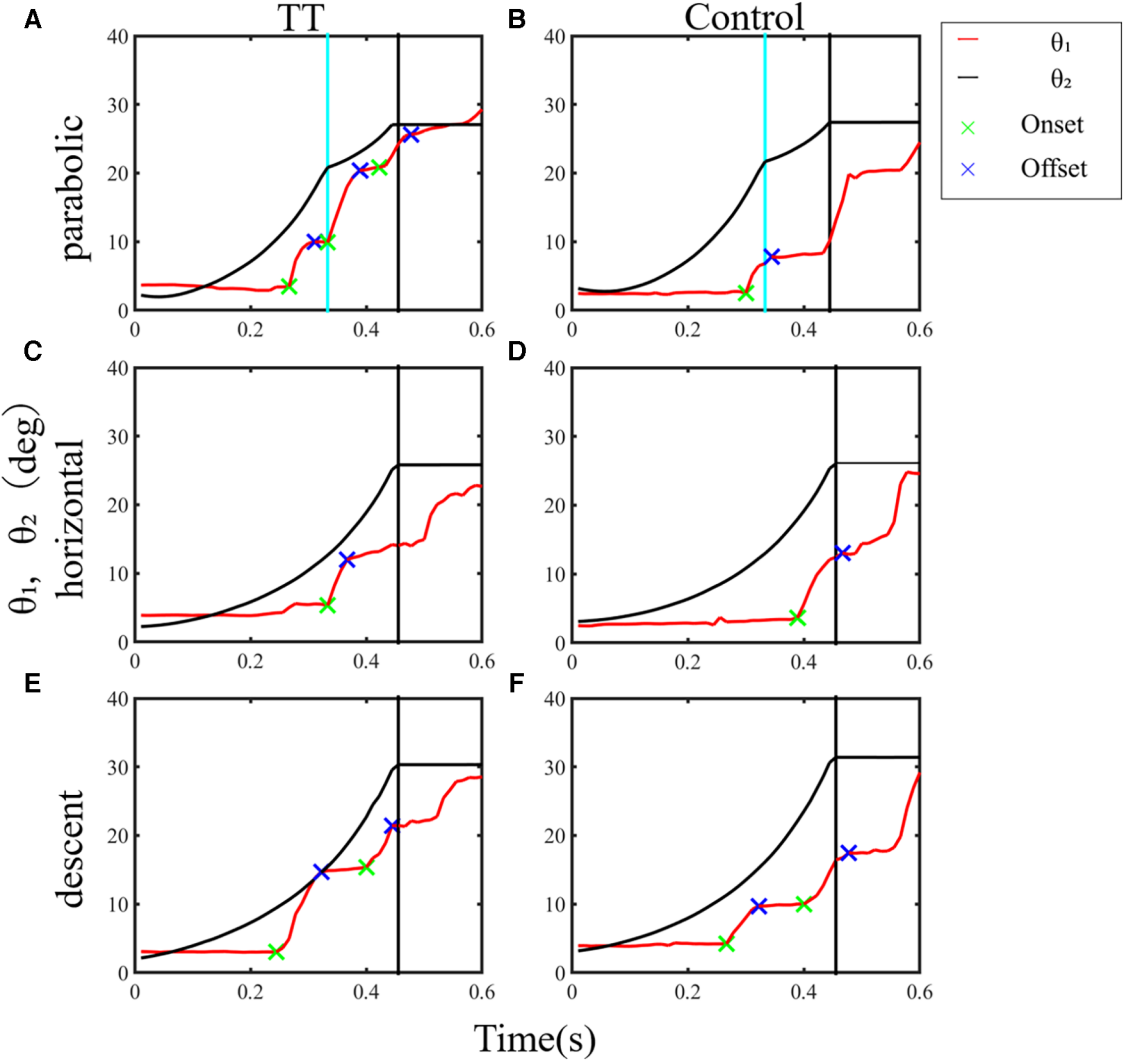
Typical examples of temporal changes in the gaze direction and ball position in a trial from a participant for each group. (**A**) Parabolic motion condition. (**B**) Horizontal motion condition. (**C**) Descent motion condition. Redline (*θ*₁): gaze direction; green and blue cross marks: onset and offset times of the saccade, respectively; blackline (*θ*_2_): ball position; black vertical line: the end of the trial when the ball reached 10 cm from the front edge of the table; pale blue vertical line: time for the ball to contact the table.

In this study, we validated the working hypothesis that table tennis players have superior gaze-tracking ability for a moving visual target by comparing ball-tracking task performance between the TT group and the Control group. Figure 3 presents typical examples of temporal changes in gaze direction and ball position during a trial of the ball-tracking task at a speed of 0.8 for one participant from each group. The TT and Control groups primarily used saccadic eye movements to track the moving ball. The first saccade occurred approximately 300 ms after the ball started to move, directed the gaze to reach the ongoing target (catch-up saccade), and multiple saccades occurred thereafter. Since the start position of the second and subsequent saccades differed depending on the end position of the previous saccade, it was difficult to analyze the spatiotemporal parameters of all other saccades on the same basis. Hence, we analyzed the catch-up saccade observed first in trials when multiple saccades occurred during a trial in this study.

#### 3.1.1 Saccade occurrence rate and smooth pursuit occurrence rate

**Figure 4:**
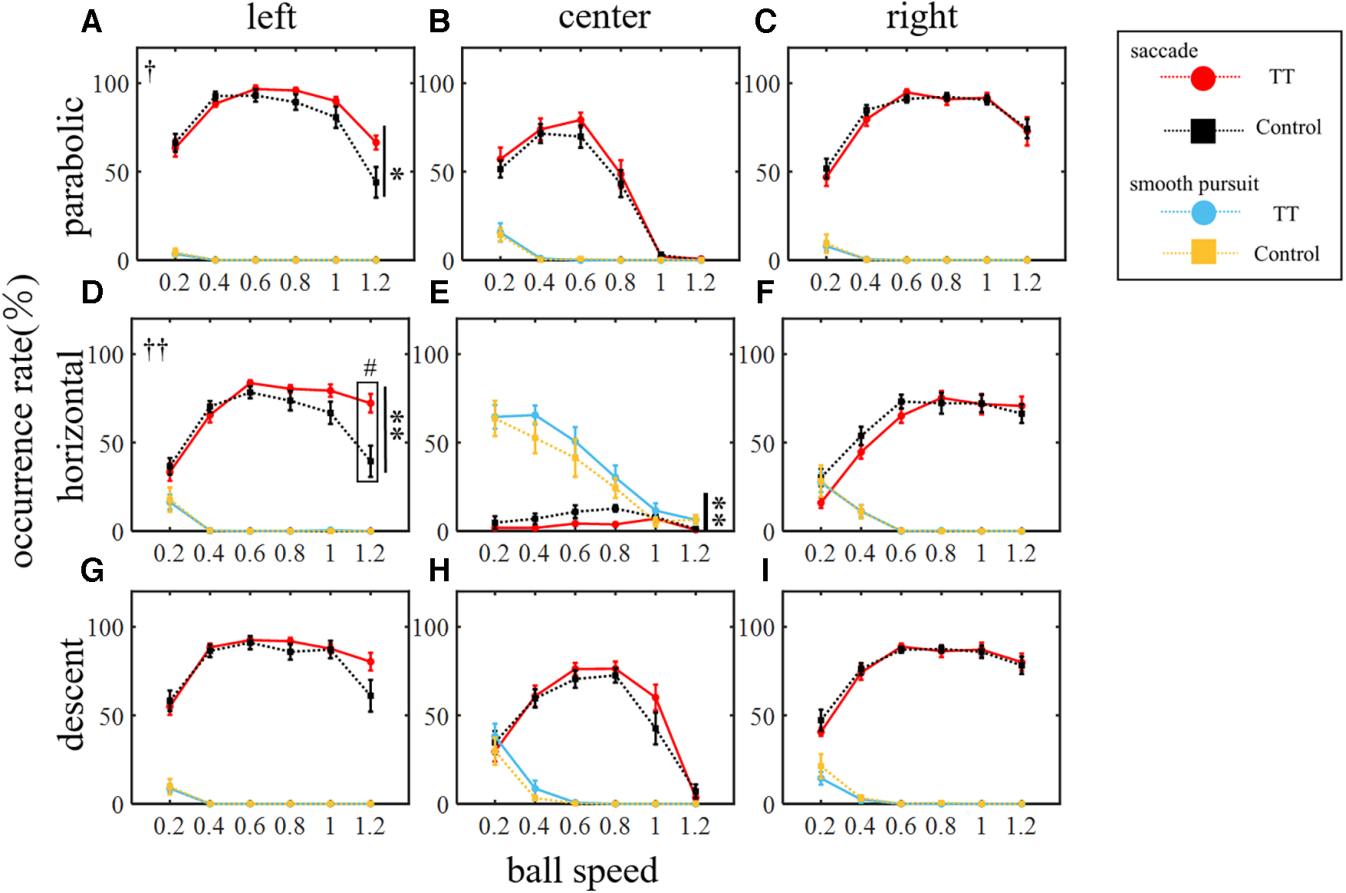
Occurrence rate of saccadic and smooth pursuit eye movements. The rows represent the trajectory of the ball: (from top) parabolic motion (**A–C**), horizontal motion (**D–F**), and descent motion (**G–I**). The columns represent the course of the ball: (from left) left (**A,D,G**), center (**B,E,H**), and right (**C,F,I**) courses. Vertical lines with symbols indicate statistical significance between groups. The red and blue circles indicate the TT group and the black and orange squares indicate the Control group. Statistically significant differences in the comparison between the TT and Control groups were observed only for saccades and not for smooth pursuits. **p < 0.01 TT vs. Control, ^†^*p* < 0.05 Interaction, ^††^*p* < 0.01 Interaction. ^#^*p* < 0.05 TT vs. Control; post-hoc test.

We investigated the saccade occurrence rate which is defined as the ratio of trials in which at least one saccade occurred to the total number of trials. Figure 4 shows the average saccade occurrence rate of all participants for each group plotted against ball speed. The saccade occurrence rate was analyzed against the ball speed for each trajectory and course (Figure 4). A two-way ANOVA showed that the main effect of ball speed was significant for all trajectories and courses (*p* < 0.01), but that of the group differed depending on the ball trajectory and course. The main effect between groups was significant for the left course of the parabolic motion condition and for the left and center courses of the horizontal motion conditions (left in parabolic: *p* < 0.05; left in horizontal: *p* < 0.01; center in horizontal: *p* < 0.01) but not for the other conditions (center in parabolic: *p* = 0.20; right in parabolic: *p* = 0.58; right in horizontal: *p* = 0.14; left in descent: *p* = 0.10; center in descent: *p* = 0.30; right in descent: *p* = 0.70). A significant interaction was observed for the left course of the parabolic motion condition (*p* < 0.05) and the left course of the horizontal motion condition (*p* < 0.01) but not for the other conditions (center in parabolic: *p* = 0.93; right in parabolic: *p* = 0.89; center in horizontal: *p* = 0.29; right in horizontal: *p* = 0.29; left in descent: *p* = 0.20; center in descent: *p* = 0.36; right in descent: *p* = 0.86). The post-hoc tests in conditions where the interaction was significant showed that the TT group was significantly higher than the Control group at a speed of 1.2 (*p* < 0.05) in the left course of the horizontal motion condition and there was no significant difference in the parabolic motion condition at any ball speed including a speed of 1.2 (*p* = 0.18). In the descent motion condition, no significant between-group differences were observed in any course, and no interactions were observed. Thus, the saccade occurrence was found to depend on the ball speed, the course, and the trajectory conditions. In the central course of the horizontal motion condition, the ball moved straight toward the participant's head. Therefore, the eye movement speed required to track the ball was less than 15 deg/s in this condition, although it was more than 20 to 40 deg/s in other conditions. For this reason, the saccade occurrence rate was extremely low compared to other conditions and was detected as an outlier from other conditions by the Smirnov–Grubbs test. Therefore, the central course of the horizontal motion condition was excluded from the following saccade-related analyses in this study.

**Figure 5:**
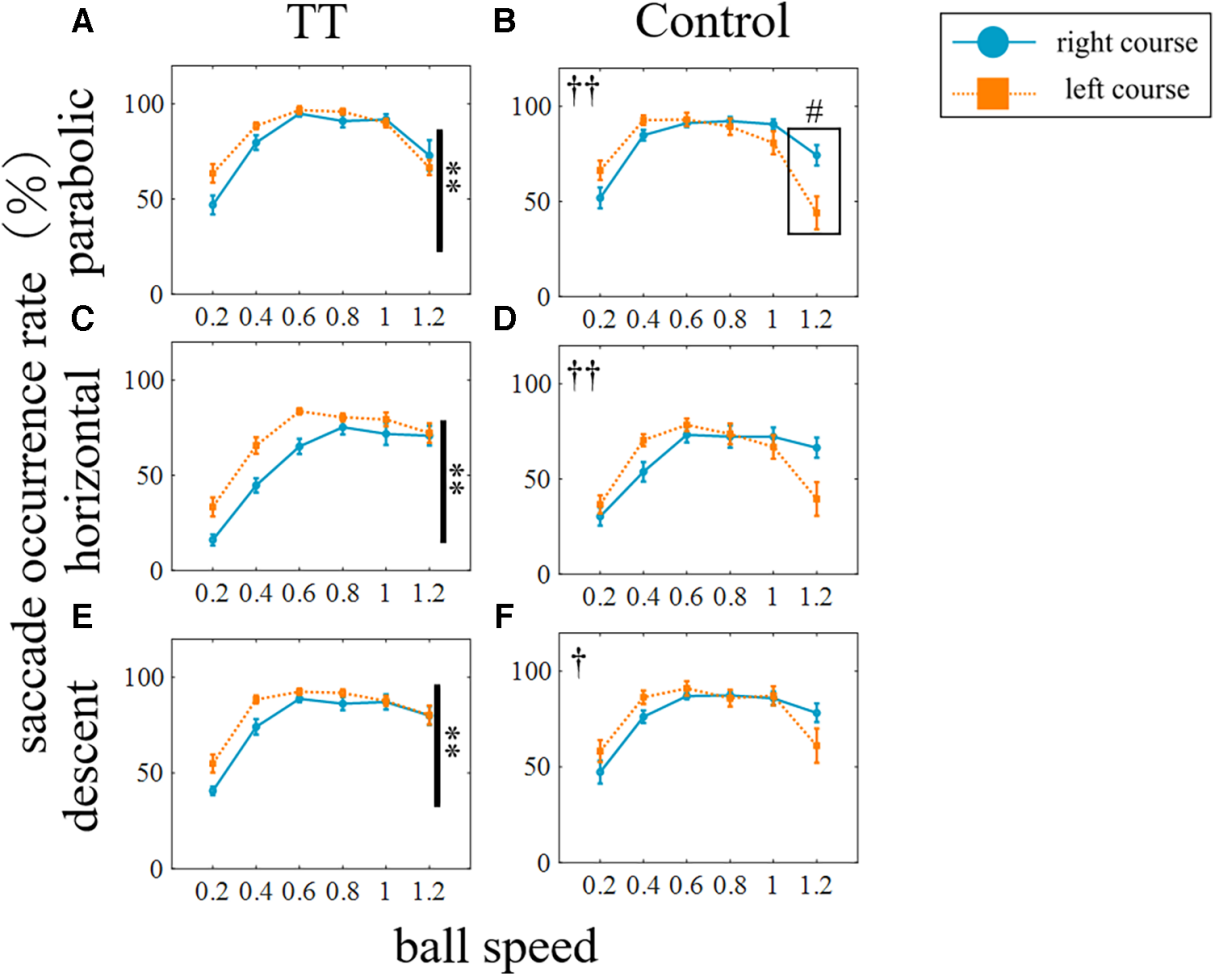
Saccade occurrence rates for left and right courses. The rows represent the trajectory of the ball: (from top) parabolic motion (**A,B**), horizontal motion (**C,D**), and descent (**E,F**) motion. The columns represent the group: (from left) TT and Control. The blue circle indicates the right courses, the orange square indicates the left courses. Vertical lines with symbols indicate statistical significance between the right course and the left course. **p < 0.01 right course and left course, ^†^*p* < 0.05 Interaction, ^††^*p* < 0.01 Interaction. ^#^*p* < 0.05 right course and left course; post-hoc test.

Since course-dependent differences between groups were observed under the parabolic and horizontal motion conditions only, we compared the saccade occurrence rates for each group between the right and left courses (Figure 5). We found that for the TT group, the saccade occurrence rate was significantly higher on the left course than on the right course in all trajectories (*p* < 0.01), but no interaction with speed was observed. In the Control group, no main effect was observed between the right and left courses, but an interaction was observed for all trajectories (parabolic and horizontal: *p* < 0.01; descent: *p* < 0.05). At the speed of 1.2 in the parabolic motion, the saccade occurrence rate on the left course was significantly lower than on the right course (*p* < 0.05). Thus, the saccade occurrence rate differed depending on the left and right direction of saccades even within the same group, and the left-right difference also differed depending on whether or not the participants had table tennis experience.

The occurrence rate of smooth pursuits was analyzed against the ball speed for each trajectory and course (Figure 4). A two-way ANOVA showed that the main effect of ball speed was significant for all trajectories and courses (*p* < 0.01) but the main effect between groups was not significant (left course in parabolic: *p* = 0.74; center in parabolic: *p* = 0.85; right in parabolic: *p* = 0.86; left in horizontal: *p* = 0.92; center in horizontal: *p* = 0.13; right in horizontal: *p* = 0.96; left in descent: *p* = 0.85; center in descent: *p* = 0.22; right in descent: *p* = 0.32). No significant interactions were also observed (left course in parabolic: *p* = 0.99; center in parabolic: *p* = 1.00; right in parabolic: *p* = 1.00; left in horizontal: *p* = 1.00; center in horizontal: *p* = 0.92; right in horizontal: *p* = 1.00; left in descent: *p* = 1.00; center in descent: *p* = 0.71; right in descent: *p* = 0.64). Thus, the occurrence rate of smooth pursuits did not differ between the TT and Control groups in any condition.

#### 3.1.2 Saccade onset time

**Figure 6:**
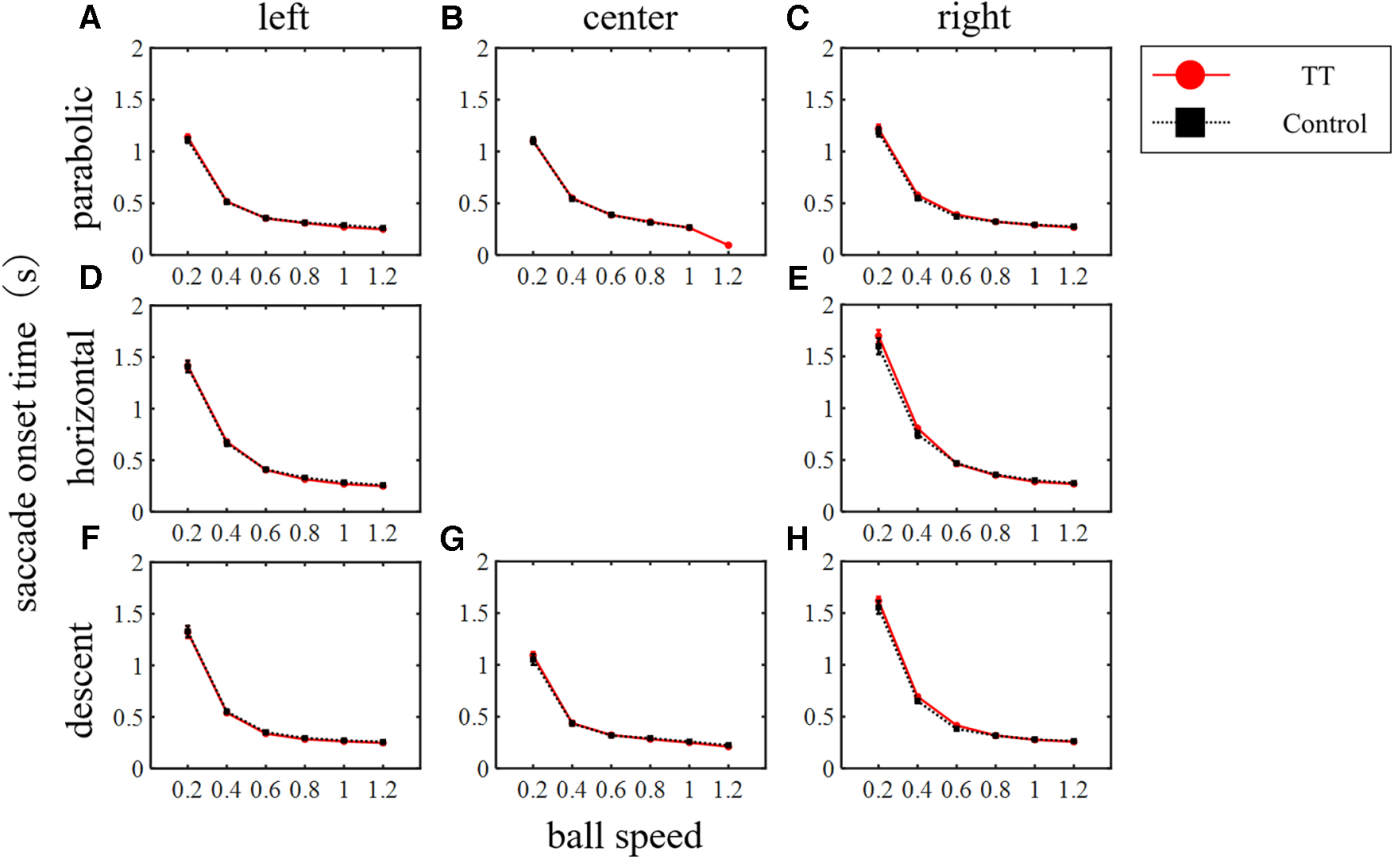
Saccade onset time. The main effect of the ball speed was significant in all trajectories and courses (*p* < 0.01) but no difference between groups was observed (two-way ANOVA).

The reaction time from the start of the target movement to the generation of the saccade was evaluated as the saccade onset time (Figure 6). The saccade onset time decreased as the ball speed increased irrespective of the ball trajectory or course, reaching about 0.3 s. A two-way ANOVA showed that the main effect of the ball speed was significant for all trajectories and courses (*p* < 0.01) but the main effect of the group was not (left course in parabolic: *p* = 0.94; center in parabolic: *p* = 0.64; right in parabolic: *p* = 0.33; left in horizontal: *p* = 0.31; right in horizontal: *p* = 0.54; left in descent: *p* = 0.48; center in descent: *p* = 0.65; right in descent: *p* = 0.11). Thus, the motor command generation time for saccadic eye movements depended on ball speed, but there was no difference between groups.

#### 3.1.3 Saccade speed

**Figure 7:**
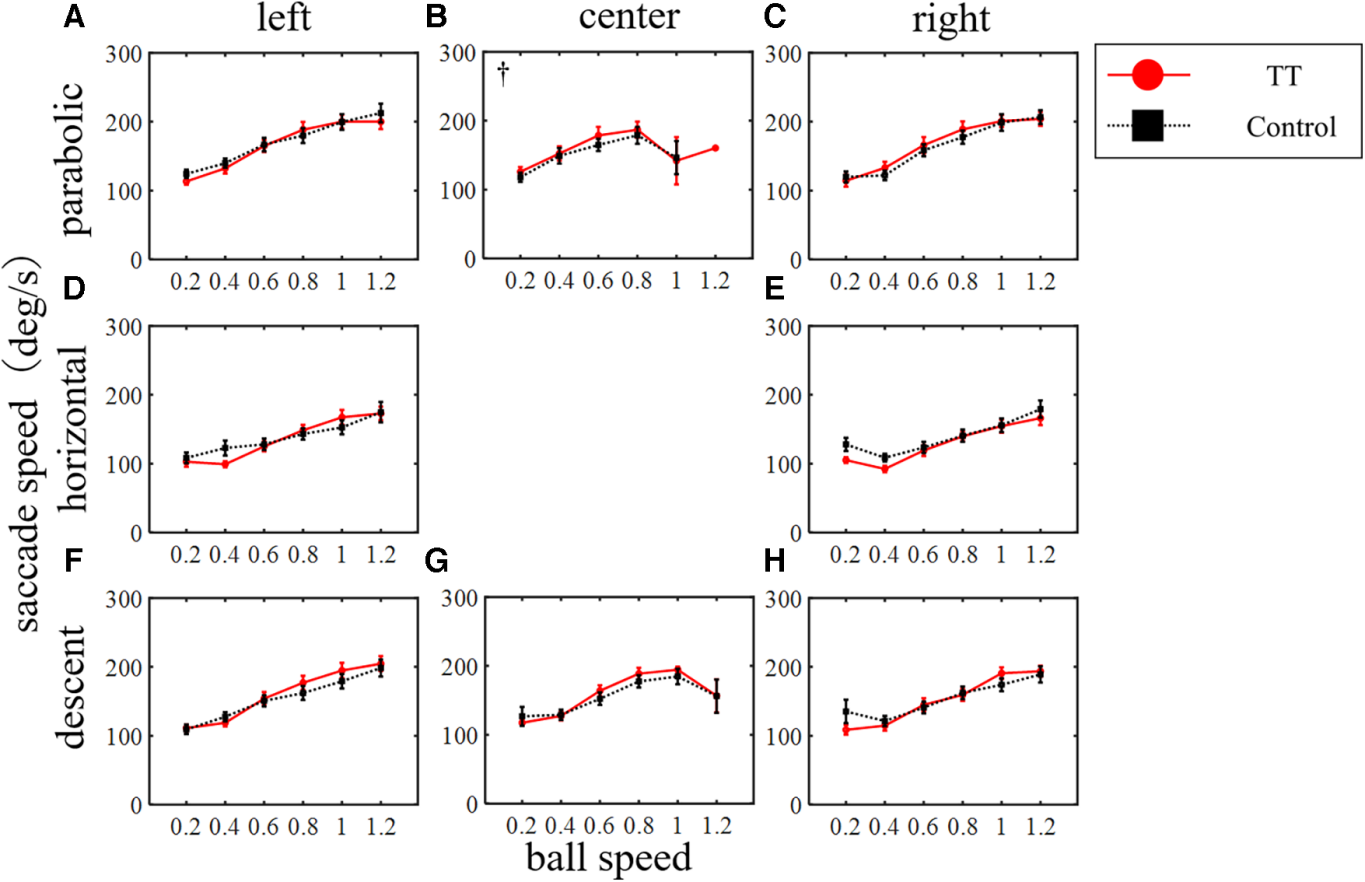
Saccade speed. The main effect of the ball speed was significant in all trajectories and courses (*p* < 0.01) but no difference between groups was observed (two-way ANOVA). ^†^*p* < 0.05 Interaction.

The saccade speed, which is a factor in determining ball tracking performance, increased with ball speed for both the TT group and the Control group regardless of ball trajectory or course (Figure 7). No significant difference was observed in the saccade speed between the two groups across all trajectories and courses (left course in parabolic: *p* = 0.82; center in parabolic: *p* = 0.81; right in parabolic: *p* = 0.53; left in horizontal: *p* = 0.85; right in horizontal: *p* = 0.45; left in descent: *p* = 0.14; center in descent: *p* = 0.26; right in descent: *p* = 0.31; two-way ANOVA). An interaction between the groups and the ball speed was observed only in the center course of the parabolic motion condition (*p* < 0.05). Thus, saccade speed depended on ball speed, but there was no difference between groups.

#### 3.1.4 Saccade endpoint error

**Figure 8:**
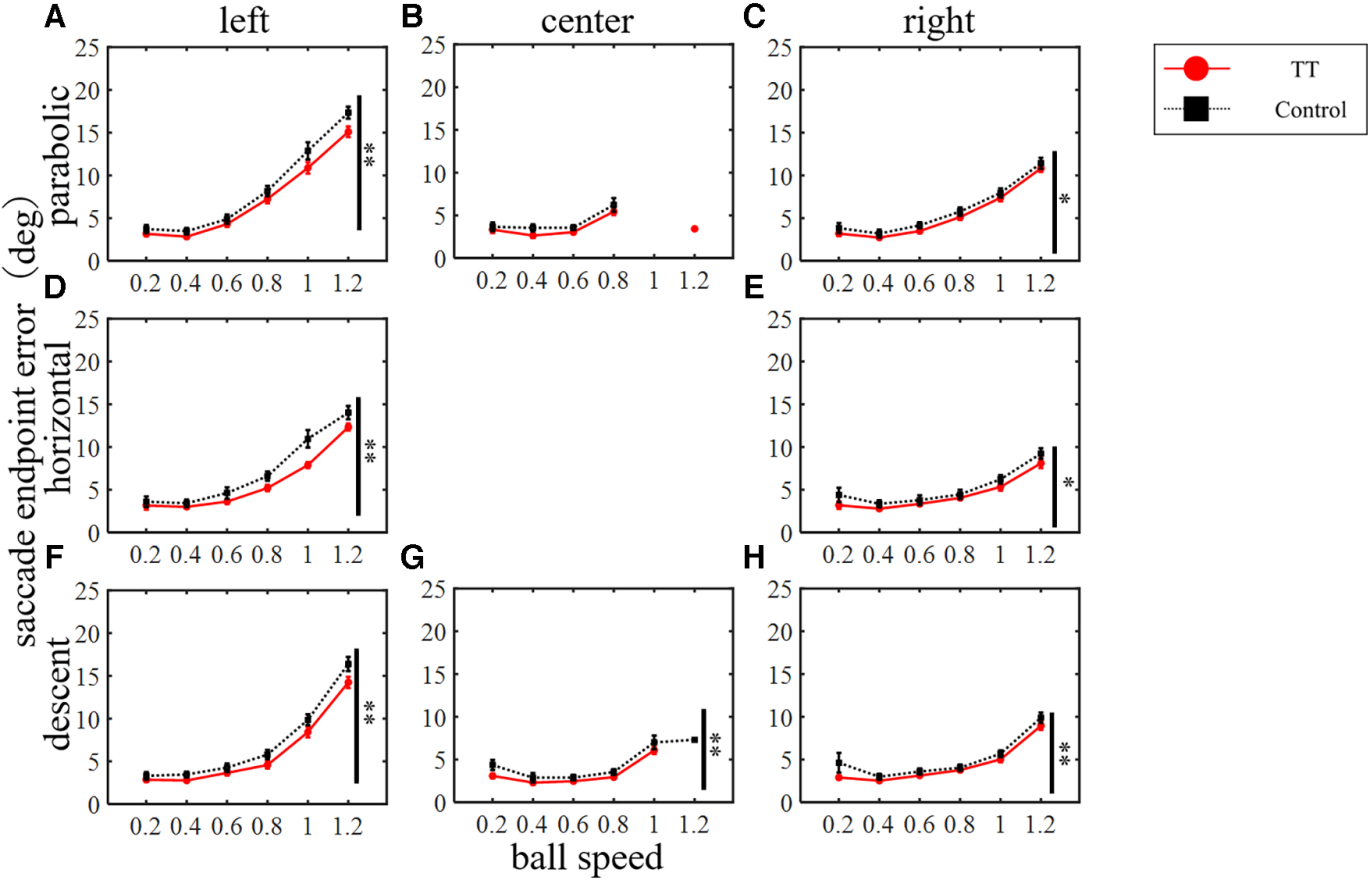
Saccade endpoint errors. Vertical lines with symbols indicate statistical significance between groups. *p < 0.05 TT vs. Control, **p < 0.01 TT vs. Control.

The saccade endpoint error was evaluated as the spatial accuracy and increased with increasing target speed in both groups (Figure 8). A two-way ANOVA revealed a statistically significant difference between the groups depending on the course of the ball but not on the trajectory (left course in parabolic: *p* < 0.01; right in parabolic: *p* < 0.05; left in horizontal: *p* < 0.01; right in horizontal: *p* < 0.05; left, center, and right in descent: *p* < 0.01). No significant interaction was observed (left course in parabolic: *p* = 0.64; center in parabolic: *p* = 0.73; right in parabolic: *p* = 0.99; left in horizontal: *p* = 0.10; right in horizontal: *p* = 0.97; left in descent: *p* = 0.76; center in descent: *p* = 0.41; right in descent: *p* = 0.69; two-way ANOVA). Thus, the saccade endpoint error had little dependence on the ball motion trajectory or course, and the TT group had small error values in all conditions except for the center course of the parabolic motion condition.

**Figure 9:**
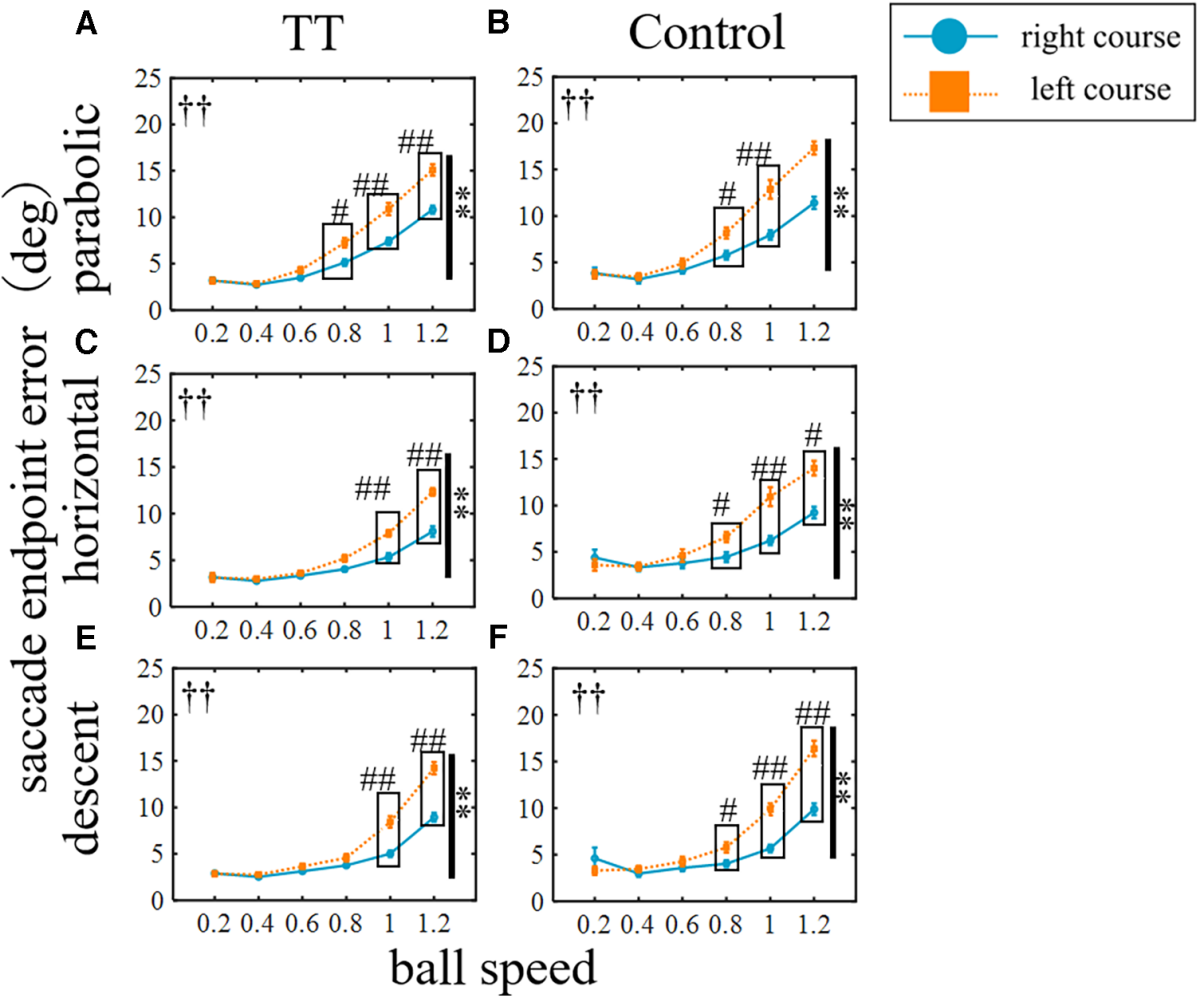
Saccade endpoint errors for left and right courses. The blue circle indicates the right courses, the orange square indicates the left courses. Vertical lines with symbols indicate statistical significance between the right and left courses. **p < 0.01 right course vs. left course, ^††^*p* < 0.01 Interaction. ^#^*p* < 0.05 right course vs. left course, ^##^*p* < 0.01 right course vs. left course; post-hoc test.

The saccade endpoint error was also compared between the right and left courses for each group (Figure 9). The error for the left course was significantly larger than that for the right course in all trajectories for both the TT and Control groups (*p* < 0.01). Post-hoc tests showed significant between-course differences in the TT group for the parabolic trajectory at speeds of 0.8, 1, and 1.2, for the horizontal trajectory at speeds of 1 and 1.2, and for the descent trajectory at speeds of 1 and 1.2.

**Figure 10:**
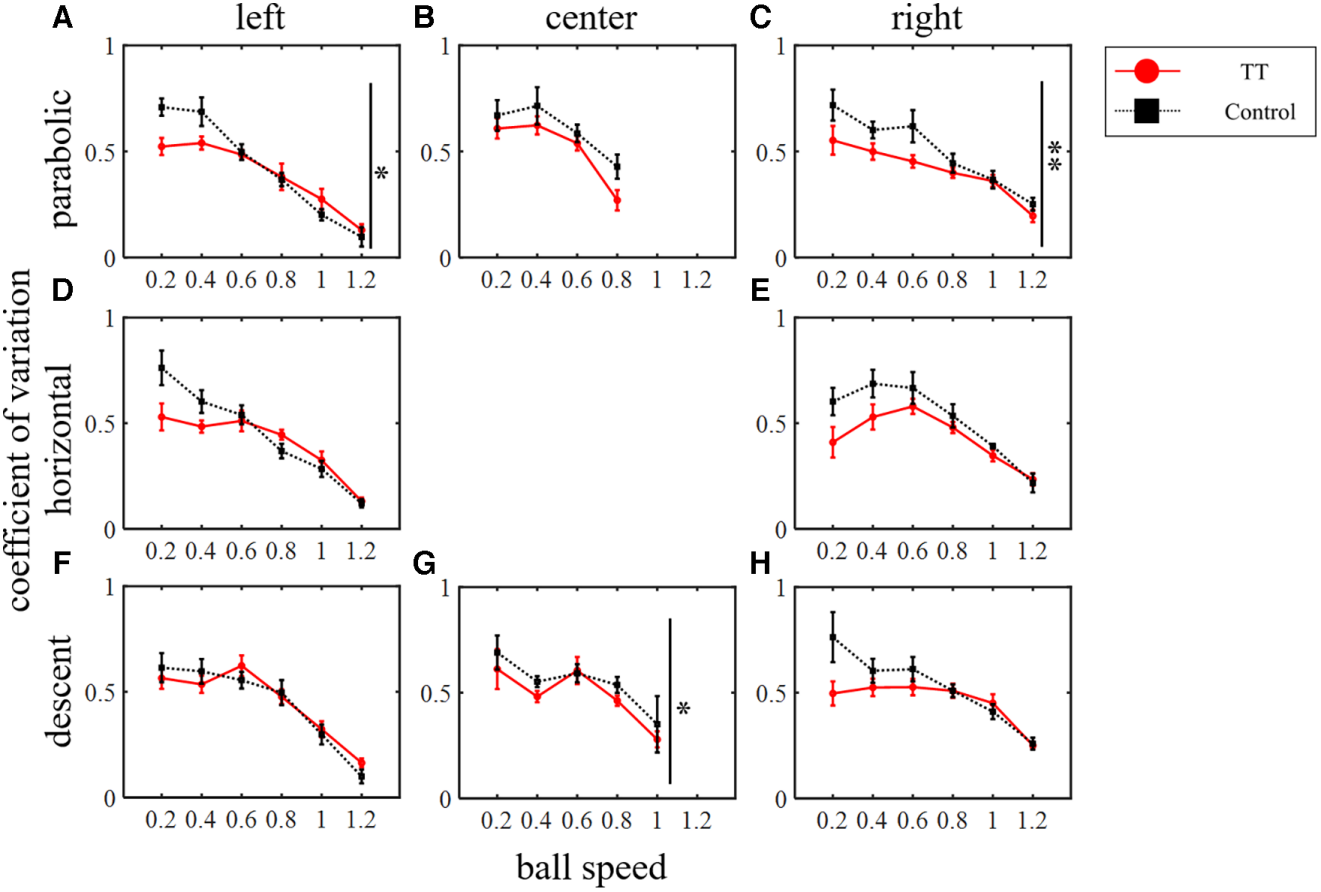
The coefficient of variation of saccade endpoint error. Vertical lines with symbols indicate statistical significance between groups. *p < 0.05 TT vs. Control, **p < 0.01 TT vs. Control.

The TT group repeated saccades to moving targets countless times during their play, so their saccades may not only have higher spatial precision but also greater consistency and reproducibility of movement across trials. Therefore, we investigated the variation coefficient of saccade endpoint error as the precision of saccade (Figure 10). In the parabolic motion condition, the main effect between groups was significant for the left and right courses (left: *p* < 0.05; right: *p* < 0.01; Friedman's test) but not for the central course (*p* = 0.46). In the horizontal motion condition, there was no main effect between groups (left: *p* = 0.09; right: *p* = 0.11; Friedman's test). In the diagonal downward motion condition, there was a significant difference between the groups at the center of the course (center: *p* < 0.05; Friedman's test), and no significant difference was observed between the right and left courses (left: *p* = 0.94; right: *p* = 0.20).

This experiment was conducted over three days, so it was necessary to confirm that no learning effects occurred during that time. Since participants performed 20 trials for each condition, we compared the results of saccade endpoint error between the first 10 trials and the second 10 trials for each condition. Neither the TT group nor the Control group were significantly different in their comparisons: for the TT group (left course in parabolic: *p* = 0.32; center in parabolic: *p* = 0.68; right in parabolic: *p* = 0.08; left in horizontal: *p* = 0.55; right in horizontal: *p* = 0.32; left in descent: *p* = 0.32; center in descent: *p* = 1.00; right in descent: *p* = 0.09), and for the Control group (left course in parabolic: *p* = 0.13; center in parabolic: *p* = 0.07; right in parabolic: *p* = 0.32; left in horizontal: *p* = 0.55; right in horizontal: *p* = 1.00; left in descent: *p* = 0.23; center in descent: *p* = 0.35; right in descent: *p* = 0.57).

### 3.2 Experiment 2

**Figure 11:**
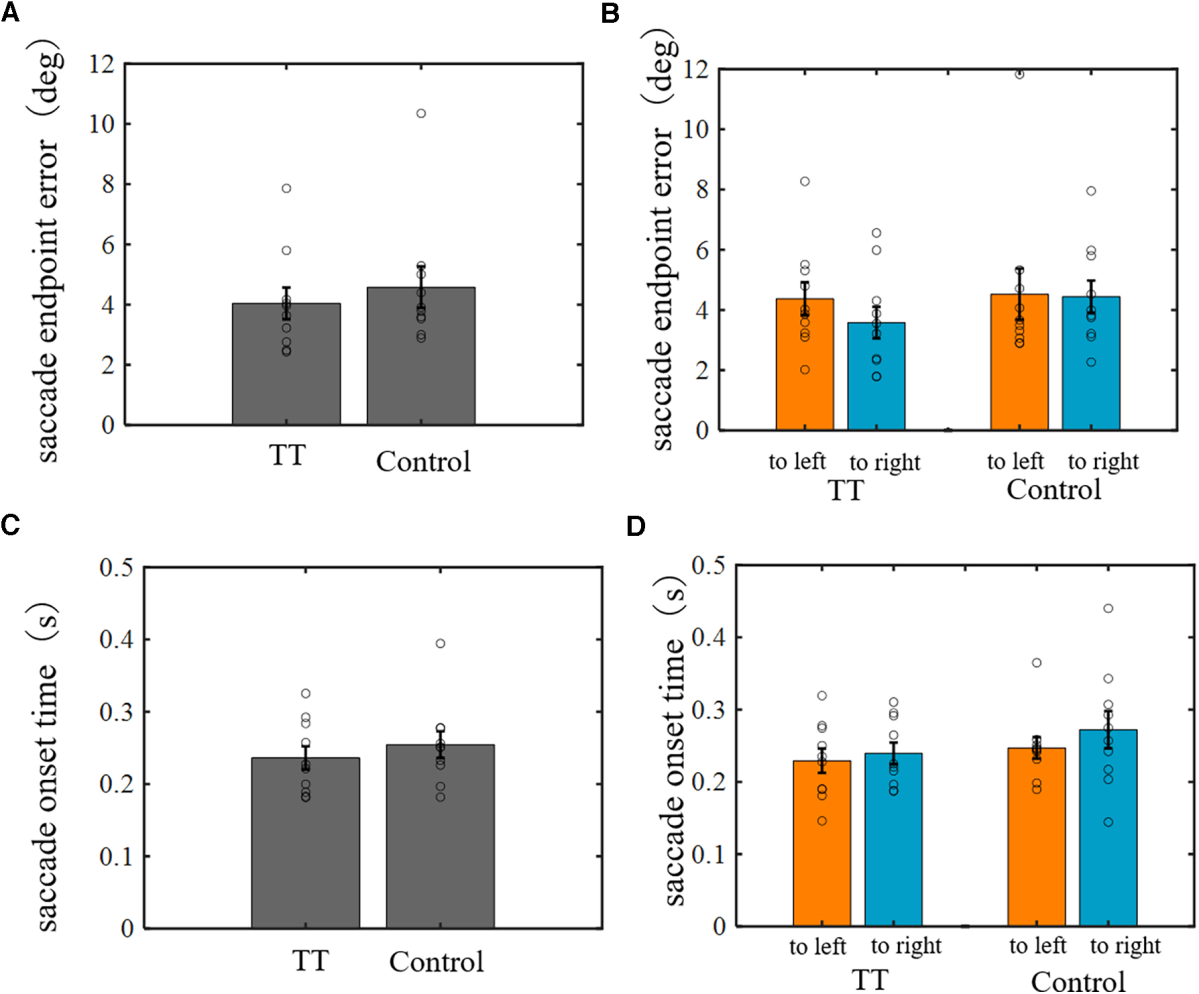
The endpoint error and onset time of saccades to stationary targets. (**A,C**) Comparison between groups. (**B,D**) Comparison between saccade directions in each group. No significant differences were found in either endpoint error or onset time between groups or saccade directions.

Experiment 1 showed that the TT group saccades had better spatial accuracy for moving targets. This finding suggests that table tennis players may have a better ability to predict the future position of the ball and/or better oculomotor control to execute saccades to the predicted position. Saccades to a moving target are thought to require information processing of both the position and motion velocity of the target at a certain point in time. Therefore, to understand which information processing of the saccade system is superior, we examined the saccade ability for stationary targets, which do not require motion information processing. We measured the endpoint error and onset time of saccades to stationary targets presented at random positions 30 cm above the table tennis table (Figure 11). No significant differences between groups were observed for either the saccade endpoint error or onset time (one-way ANOVA). This suggests that table tennis players are better able to predict the future position of the ball.

Additionally, in Experiment 1, the saccade ability for a moving target between the left and right courses was significantly different. Therefore, we analyzed saccades for a stationary target for the left and right directions separately but again found no significant difference in saccade endpoint error or onset time for each group.

## 4 Discussion

The results of this study are summarized as follows: (1) both the TT and Control groups tracked a ball that began to move from rest using catch-up saccadic eye movements; (2) the TT group had a better saccade ability for moving targets (occurrence rate, spatial accuracy, and inter-trial precision); (3) the superiority of the occurrence rate of the TT group was observed only in the high ball speed range on the left course; (4) the TT group had a high precision of the saccade endpoint error in the parabolic trajectory; and (5) the TT group had no superiority for saccades to stationary targets. These results support our hypothesis that table tennis players have a superior ability of saccades to capture moving targets as a result of stimulus-specific improvements in motion vision by observing the ball's myriad movements.

### 4.1 Superior ball-tracking ability with saccadic eye movements highlights the importance of saccadic gaze behavior in table tennis

Studies on gaze behavior in table tennis players have shown that they focus on the ball immediately after contact with the racket of the opponent (8, 23, 24). It is where the ball's destination is most uncertain because subtle changes in the angle of the racket surface have a large effect on the direction of the ball's travel, and speed and trajectory also change dramatically. Table tennis players' actions begin with quickly and accurately determining the direction and speed of the ball struck by their opponent, and the first part of the ball's trajectory is visually tracked (23, 24). The task in this study mimics this situation.

The gaze behavior of directing one's gaze to where the ball is released has been observed not only in table tennis but also in sports to intercept high-speed balls, such as baseball (25) and cricket (26, 27). Batters in these sports are under severe time constraints and must plan the appropriate motor response based on visual movement information as early as after the ball release to secure time for batting movements (8). It is known that tracking the released ball with a smooth pursuit improves ball prediction accuracy (28, 29) and interception accuracy (30, 31). Consistent with this, it has been reported that expert baseball and cricket players use smooth pursuit to look at the ball released for longer periods than unskilled players (26, 32, 33). The ball-tracking by smooth pursuit is useful in sports where pitchers have limited directions in which to throw (e.g., toward the home plate or wicket). This is because the eye and head movements with a large angular velocity are not required to track the ball immediately after it has been pitched (4). However, in the case of table tennis, where high-speed balls are launched in various directions from an opponent approximately 3 meters away, saccades are essential, as high-speed eye movements are required that go beyond the tracking ability of smooth pursuit. Supporting this, not only the control group but also the table tennis players tracked the ball using saccades in this study. Therefore, ball-tracking through saccades is essential in table tennis, and its spatial accuracy is assumed to contribute to hitting performance.

In sports where the ball bounces, such as table tennis or cricket, saccades are used to direct the gaze ahead of the ball to the point where it is expected to bounce (8, 23, 24, 26). Land et al. (2000) compared skilled and unskilled cricket batsmen and reported that the higher-level batsmen made earlier and more accurate predictive saccades. This means that skilled experts can use the information obtained immediately after ball release to predict bounce events more accurately and in advance. In this study, table tennis players could make saccades with high spatial accuracy to a ball that started moving from their fixation point. This suggests that, like expert cricket players, table tennis players can better analyze the motion of their moving targets and predict where they will reach in the future.

Table tennis players make several saccades to track the ball when the ball speed is low (8). Multiple saccades were also observed at low ball speeds in this study, but at medium to high ball speeds (0.8, 1, 1.2), there is not much time to generate a second saccade. In such situations, the striking performance of players depends on whether or not they can generate saccades based on visual information immediately after the opponent's striking, and whether they can generate saccades with high accuracy. The table tennis players in this study showed superiority in occurrence rate and spatial accuracy of saccades even in the ball high-velocity range, suggesting that these abilities are required in table tennis and that they can be improved through table tennis itself as training.

Saccades quickly shift the eyes to a new location with limited online movement control. Therefore, if there is a deviation in the arrival of a saccade to the target point, it is known that a corrective saccade will follow (34). However, it has been reported that the corrective saccades change the speed perception and arm's intercept action to a moving target depending on the saccade direction (35), which is expected to have undesirable effects on athletes. Therefore, improving the saccadic ability attracted by this study, namely, the incidence and spatial accuracy of saccades occurring after an opponent strikes, will lead to improved striking performance in table tennis.

### 4.2 The superior saccade ability of table tennis players may reflect the superiority of brain information processing pathways relevant for moving targets

Notably, the TT group showed a superior saccade ability to track moving targets, but the two groups had comparable saccade ability to track stationary targets. This result suggests that the repeated experience of saccade-tracking a fast-moving ball during table tennis trained the associated saccade-generating mechanism and induced plastic changes. The repetitive practice of skill-specific body movements is very effective in acquiring and improving specific motor skills in sports, and the plastic reorganization that occurs in the neural circuits used during this repetition that essential for the improvement (22). Similarly to general motor skills, eye movement ability undergoes plastic changes with practice (36, 37). For example, Dyckmank and McDowell (38) investigated the effect of training on three different eye movement tasks (antisaccade, prosaccade, and fixation) and found that antisaccade performance was improved by antisaccade training, prosaccade task diminished antisaccade performance, and fixation training did not affect antisaccade performance. Therefore, the direction and strength of the training effect on each type of eye movement will differ depending on the type and content of the training to be undertaken. The effect of training on eye movement has been attributed to different cerebral cortical regions and neural circuits for different types of saccades (39, 40). These previous studies suggest our observations are the result of changes in specific brain regions caused by playing table tennis.

In humans (41), the middle temporal complex (MT) of the dorsal visual pathway in the cerebral cortex processes visual motion information and supplies motion signals to various networks that require target motion information. In monkeys (42), disruption of the MT by ibotenic acid impairs pursuit and saccades against moving targets, resulting in large saccade endpoint errors. However, saccades to stationary targets were not affected by this lesion, indicating that both target position detection and saccadic motor control for accurate landing on the target were intact. The loss of the MT impaired motion information processing, compromising the accuracy of the saccade amplitude estimation. In humans too, the accuracy of motion information processing in the MT contributes to the spatial accuracy of the saccade for the moving target (43).

Human and monkey studies have reported that activity in the MT is closely related to the velocity of moving targets. For example, the reaction time required to detect a target motion and initiate body movement decreases non-linearly with increasing target motion speed. Consistent with this relationship, the latency of neuron-related magnetic response in the occipitotemporal region, including the human MT, also shortens non-linearly with increasing target movement speed (44). Similar properties have been reported for the response latency of the N2 component of the motion-evoked potential recorded in the human MT, which is thought to reflect information processing related to motion perception (45). These previous findings indirectly suggest that a shortened response latency in the human MT leads to a shortened physical response time. Similar results were also observed in the present study, where the onset time of the saccade eye movement decreased non-linearly with increasing target movement speed. Since the visual information to generate a saccade in response to a moving target is 100 ms before the start of the saccade (46), we calculated the angular velocity from data 100 ms before the onset of the saccade. For parabolic motion, target motion speeds of 0.2, 0.4, 0.6, 0.8, 1, and 1.2 corresponded to approximately 20, 36, 42, 69, 97, and 121 deg/s, respectively. Similarly, for the horizontal motion condition they were 24, 30, 33, 42, 53, and 61 deg/s, respectively, and for the descending motion they were 17, 16, 30, 42, 57, and 78 deg/s, respectively. Kawakami et al. (44) measured magnetoencephalographic (MEG) neural responses in the occipitotemporal region to the motion initiation of light over a wide range of velocities (0.4–500 deg/s) in humans and measured the physical reaction time. Both the reaction time and MEG response latency were inversely proportional to the target velocity. In particular, significant shortening occurred between target velocities of 0.4 and 30 deg/s. A similar target velocity-MEG response latency function was also reported by Maruyama et al. (2002) (47). In the present study, a significant reduction in the saccade onset time was observed for velocity conditions between 0.2 and 0.6 for all ball trajectories, which approximate 2–30 deg/s. This result agrees with the relationship between the body's reaction time and target motion speed in previous studies (48, 49). Considering the results of Kawakami et al. (2002) and Maruyama et al. (2002), the motion information in the human MT may be used not only for physical reactions, such as button pressing, but also for catch-up saccades, and the information provision speed may be a temporal rate-limiting factor for visuomotor responses.

The superiority of the saccade spatial accuracy (endpoint error) by the TT group may be linked to the human MT. In both groups, the error increased non-linearly when the ball speed increased above the condition of 0.6. Between speeds of 0.6 and 1.2, the ball velocity increased from about 30 deg/s to 100 deg/s. It is known that neurons in monkey MT have tuning properties for motion target velocity, with most neurons exhibiting optimal responses at a velocity of 4–16 deg/s (50, 51). An fMRI study showed that neurons in the human MT have an optimal response of 7–30 deg/s (52). Therefore, the target speed-dependent increase in endpoint error observed in the present study at velocity conditions of 30 deg/s or higher is possibly due to inaccurate information provision by exceeding the optimal motion velocity condition for the motion analyzer in the human MT. If so, the superiority of the saccade spatial accuracy (endpoint error) in the TT group in the high-speed condition was possibly brought about by an improved motion analysis ability in the human MT. Since a motor command of the saccade to the moving target cannot be generated without motion information of the target, the decrease in the rate of the saccade occurrence rate in high-speed conditions on the left course might be due to the same reason. Further research on this point is needed.

### 4.3 Exposure experience with visual motion stimulation during table tennis may have improved the visual system of the players

Table tennis players are exposed to visual motion stimuli at high frequency and for long periods. When the visual system is exposed to a specific visual stimulus for a long time or frequently, its detection sensitivity to the exposed stimulus improves through plastic changes called perceptual learning (15). It has been reported that prolonged motion stimulation improves perceptual motion detection and discrimination abilities (16–19). Therefore, table tennis players may have an improved ability to analyze the motion of moving targets, which contributes to the superiority of the saccade ability observed in this study. Learning effects in perceptual learning are known to be specific to the exposed stimulus (15), and the above-mentioned improvement effects on perceptual motion detectability and discriminability are specific to the exposed stimulus direction (16, 17).

The superiority of the saccade spatial accuracy of the TT group was observed not only for parabolic trajectories, which are commonly seen in table tennis rallies, but also in the horizontal and descent motion trajectories, which have no equivalent in table tennis rallies. However, we believe that our results are consistent with the stimulus specificity of perceptual learning. In previous studies, learning effects were observed even when the motion direction of the exposed stimulus was shifted by 45 degrees (16, 17). We calculated the angular difference until a saccade occurred for the parabolic motion trajectory, horizontal motion trajectory, and descending motion trajectory in this study; we found that it was only about 5 deg of the visual angle. Therefore, the learning effect caused by the most frequently seen parabolic motion may have affected other motion trajectories as well.

We found that the occurrence rate and spatial accuracy of saccades to moving targets differed depending on the course of the ball, that is, the direction of the saccade eye movement. The saccade endpoint error was significantly smaller in the right course than in the left course in both the TT group and Control group, and the difference was particularly marked at high ball speeds (see Figure 9). Such differences were not observed for other saccade parameters, and no difference was observed in rightward and leftward saccades to a stationary target, so it is unlikely to be an artifact in the measurements. It has been reported that humans have a direction-dependent bias of saccade movement. For example, when saccades were performed in the right and left directions, the onset latency was significantly longer in the left direction (53, 54). Interestingly, the reports showed that the bias was observed in right-handed individuals. There are also reports of a relationship between asymmetry and eye dominance (55). In our study, we did not precisely investigate participants' handedness and eye dominance, so the relationship between saccade lateralization bias and handedness is unclear. Another possible reason for the course difference is attention. The perceptual motion direction discriminability for the coherent motion has been reported to decrease due to divided attention, which was observed only in the left visual field, but not in the right one (56). In the present study, the participants were required to follow moving targets under 54 conditions, so it was necessary to fully utilize their attentional functions due to the high degree of uncertainty. Even small reductions in attention may decrease the generation and spatial accuracy of saccades (57). Finally, the directional bias of the saccade in daily life may be the cause. Reading horizontally written Japanese text needs a rightward saccade which is not just a saccade to a static target, but a predictive saccade that takes context into account, and has aspects in common with saccades to a moving target. The poor ability to saccade to the left in the control group may have been improved in the TT group by the repetitive leftward saccades during table tennis. Further investigation is required to clarify these points.

### 4.4 Table tennis players with better precision in saccade endpoint error hold a notable advantage in table tennis

The ability to track the ball via saccadic eye movements can be assessed not only with accuracy, defined as an absolute spatial error but also precision (or low inter-trial variability), as noted by Guthrie in 1952 (58). This study found that, specifically in parabolic motion conditions, the TT group exhibited significantly better precision in saccade endpoint error for both left and right courses compared to the Control group. This implies consistent precision in saccade execution. While trial-to-trial variation aids in learning new motor skills, it hampers reaching/interception performance (59). Hence, table tennis players with better precision in saccade endpoint error hold a notable advantage in table tennis with continuous saccades. Van Beers RJ and colleagues (2007) explored the factors influencing variation in human saccadic eye movements, identifying uncertainty in target localization as a primary factor affecting saccade endpoints (60). Consequently, this suggests a reduced uncertainty in predicting the target's future location among the TT group. Common knowledge in sports suggests that trial-to-trial variability lessens with continued practice (61, 62). The TT group's training outcomes likely reflect perceptual learning from visual exposure to motion stimuli in table tennis, with the most pronounced training effect occurring in frequently encountered parabolic motions.

### 4.5 Human saccade function is optimized in daily life but may be improved by adapting to higher needs in sports

The reason for no differences in the spatial accuracy of saccades for stationary targets between the two groups also deserves consideration. Since the central fovea of the human retina is small, everyone needs highly accurate saccades for object recognition and spatial recognition in daily life. If a spatial error occurs between the saccade arrival point and the visual target, the error is quickly corrected by motor learning, a phenomenon known as saccade adaptation. In the saccade generation mechanism for stationary targets, such a mechanism is universally present regardless of experience, which may explain the lack of difference. However, this observation does not deny the superiority of saccade ability to stationary targets in athletes, as differences have been observed between athletes and non-athletes for saccades under more complex or advanced spatiotemporal demands (9–11, 13, 63).

A superior saccade function for motor targets plays a very important role in the high performance of table tennis players. Aoyama et al. (64) demonstrated using a continuous visuomotor task that a simulated movement of a racket to a fast-moving target is amended based on the visual feedback information acquired after the saccade ends. They also showed that the spatial accuracy of the saccade determines that of the reaching movement. Accordingly, our results suggest that the improvement of the saccade spatial accuracy leads to better table tennis performance.

### 4.6 Limitations of this research and prospects

In this research, parabolic motion trajectory was reproduced faithfully in virtual space based on tracking data of a table tennis ball shot onto a table tennis table in real space. As a result, several participants of the TT group commented, “It looks so similar to the ball I see in an actual table tennis situation that almost couldn't help but want to hit it back.” Therefore, the superior ball-tracking ability achieved by saccadic eye movements found in table tennis players in this study may contribute to actual table tennis performance but the direct relationship with performance is not clear. Therefore, in future studies, it will be necessary to verify the contribution to performance by evaluating the performance of table tennis players in conjunction with their ball-tracking ability. Furthermore, the reason for the superior saccadic ability of table tennis players may reflect their superior ability to process visual motion information and the ability to predict the future position of a moving target based on visual information. However, in this study, it was not possible to distinguish between them, so future studies will be necessary to separately examine motion vision abilities, such as the ability to detect and discriminate the direction of visual motion, and predictive abilities.

## 5 Conclusions

We conclude that table tennis players have a higher ability to direct their gaze accurately and precisely at the expected arrival point of a ball moving by a catch-up saccade. The catch-up saccade performance can be improved by visual experience and visuo-ocular training executed in ball sports including table tennis, making it possible to acquire highly accurate visual information constantly, which leads to better sports performance.

## Data Availability

The original contributions presented in the study are included in the article/Supplementary Material, further inquiries can be directed to the corresponding author.
